# Watermelon juice: a promising feedstock supplement, diluent, and nitrogen supplement for ethanol biofuel production

**DOI:** 10.1186/1754-6834-2-18

**Published:** 2009-08-26

**Authors:** Wayne W Fish, Benny D Bruton, Vincent M Russo

**Affiliations:** 1 USDA-ARS, South Central Agricultural Research Laboratory, Lane, OK, USA

## Abstract

**Background:**

Two economic factors make watermelon worthy of consideration as a feedstock for ethanol biofuel production. First, about 20% of each annual watermelon crop is left in the field because of surface blemishes or because they are misshapen; currently these are lost to growers as a source of revenue. Second, the neutraceutical value of lycopene and L-citrulline obtained from watermelon is at a threshold whereby watermelon could serve as starting material to extract and manufacture these products. Processing of watermelons to produce lycopene and L-citrulline, yields a waste stream of watermelon juice at the rate of over 500 L/t of watermelons. Since watermelon juice contains 7 to 10% (w/v) directly fermentable sugars and 15 to 35 μmol/ml of free amino acids, its potential as feedstock, diluent, and nitrogen supplement was investigated in fermentations to produce bioethanol.

**Results:**

Complete watermelon juice and that which did not contain the chromoplasts (lycopene), but did contain free amino acids, were readily fermentable as the sole feedstock or as diluent, feedstock supplement, and nitrogen supplement to granulated sugar or molasses. A minimum level of ~400 mg N/L (~15 μmol/ml amino nitrogen) in watermelon juice was required to achieve maximal fermentation rates when it was employed as the sole nitrogen source for the fermentation. Fermentation at pH 5 produced the highest rate of fermentation for the yeast system that was employed. Utilizing watermelon juice as diluent, supplemental feedstock, and nitrogen source for fermentation of processed sugar or molasses allowed complete fermentation of up to 25% (w/v) sugar concentration at pH 3 (0.41 to 0.46 g ethanol per g sugar) or up to 35% (w/v) sugar concentration at pH 5 with a conversion to 0.36 to 0.41 g ethanol per g sugar.

**Conclusion:**

Although watermelon juice would have to be concentrated 2.5- to 3-fold to serve as the sole feedstock for ethanol biofuel production, the results of this investigation indicate that watermelon juice, either as whole juice fermented on-site or as a waste stream from neutraceutical production, could easily integrate with other more concentrated feedstocks where it could serve as diluent, supplemental feedstock, and nitrogen supplement.

## Background

Ethanol produced by fermentation of plant biomass is considered to be an environmentally friendly alternative to fossil fuels [1,2]. The search for renewable biomass sources that do not serve as human food and animal fodder have focused primarily on plant biomass that possesses mainly cellulose and lignocellulosic materials [3]. Although it is generally agreed that this type of biomass will ultimately be the primary source of ethanol biofuel in the future [4], there exist sources of simple hexoses that can fill a special niche in biofuel production but that have not been investigated for such an application. One such source is watermelon. Two factors make watermelon worthy of consideration as a possible feedstock for ethanol biofuel production. First, about 20% of each annual watermelon crop is rejected for fresh fruit marketing because of surface blemishes or because fruit are misshapen; these 'culls', although internally sound, are left in the field. For the 2007 growing season, this would have amounted to around 360,000 t lost to US growers as a source of revenue [5]. Conversion on the premises of all or part of these cull watermelons to ethanol could, at the very least, provide supplemental on-farm fuel to the grower, or, in some cases, be sold on the ethanol biofuel market. Second, the neutraceutical value of compounds obtained from watermelon is at a threshold whereby a portion of the watermelon crop and all the culls could be employed as starting material to extract and manufacture these products. Lycopene, the powerful antioxidant carotenoid that imparts the red color to watermelon, has been shown to be important in prostate health [6]. L-citrulline is a naturally occurring amino acid involved in the detoxification of catabolic ammonia and also serves as a precursor for L-arginine, the amino acid centrally involved in the production of the circulatory vasodilator, nitric oxide [7]. Production of these two neutraceuticals from watermelon yields a waste stream that contains ~10% (w/v) sugars that can be directly fermented to ethanol.

A watermelon is nominally 60% flesh, and about 90% of the flesh is juice that contains 7 to 10% (w/v) sugars. Thus, over 50% of a watermelon is readily fermentable liquid. A logical use for a watermelon juice processing waste stream would be integration into bioethanol production. Since it is water-based, watermelon juice could serve as a diluent for concentrated sources of fermentable sugars such as molasses that require an approximate dilution to ~25% (w/v) sugars before fermentation. In addition, the 7 to 10% (w/v) ready-to-ferment sugars in watermelon (glucose, fructose, and sucrose) would supplement the primary feedstock and reduce primary feedstock requirements proportional to the volume of watermelon juice used to dilute the concentrated feedstock. Third, the free amino acids at 15 to 35 μmol/ml in watermelon juice could serve as a nitrogen supplement for yeast in those feedstocks such as cane sugar and molasses that possess inadequate available nitrogen levels to maintain maximal yeast growth and ethanol production.

Little has been published on investigations for optimizing the parameters for fermentation of watermelon juice. Kim *et al*. [8] reported on parameters affecting the fermentation of watermelon juice for the ultimate manufacture of vinegar, but the optimization for maximal rates of ethanol production was not one of their objectives. The purpose of this investigation was to examine watermelon juice, either whole or as a waste stream from neutraceuticals production, as a diluent, feedstock supplement, and nitrogen supplement in ethanol biofuel production systems.

## Methods

### Fermentation feedstocks

Watermelons employed in this study came from several sources covering two crop years. In 2007, watermelons came from a commercial field in Hinton, OK, USA. They were purposely selected because they had been graded 'culls' as the result of an anthracnose infection on their outer rinds. Other sources of watermelons included those from the 2007 crop in Terral, OK, USA, some from the 2008 crop in Louisiana, USA, and others from the 2008 crop raised at the South Central Agricultural Research Center, Lane, OK, USA. Lycopene-free juice was a waste stream from processing watermelon flesh to produce lycopene-containing chromoplasts by a procedure previously described (Fish, US Patent Appl. 60/752.279, 2005). Amino acid-free juice was prepared from the lycopene-free watermelon juice by procedures developed in this laboratory (Fish, unpublished). Watermelon juices at the various stages of processing were frozen and stored at -20°C until used. Granulated sugar and molasses employed for fermentations were purchased at a local supermarket.

### Fermentation

Fermentations were conducted in a BF-110 benchtop modular fermentor system (New Brunswick Scientific Co., Inc., Edison, NJ, USA). The system included a 7.5 l thermostatted glass vessel, a pH/DO controller, a four-pump reagent addition module, an exhaust condenser, a DO probe, and a pH electrode. The fermentor was sanitized between fermentations by washing with detergent, rinsing with water, treating with 5% (v/v) Oxonia Active™ (Ecolab Inc., St Paul, MN, USA) for 6 h at room temperature, and rinsing thoroughly with water. Most of the experimental fermentations were conducted on a volume of 2 to 3 L of feedstock. Temperature was controlled at 32°C, and the medium stirred at 100 rpm to keep the yeast, substrate, and products evenly distributed. Media were routinely inoculated with hydrated and conditioned dried yeast to provide an initial yeast population of ~10^7 ^viable cells/ml in the fermentation medium. The pH of nearly all fermentations not pH-controlled quickly dropped to ~pH 2.8 and remained there regardless of the starting value. For those fermentations conducted at a pH other than ~3, the fermentor controller was programmed to add 1 M NaOH when needed to maintain the pH of the medium at the level desired. The starting pH of the processed watermelon juice used to prepare feedstocks was generally pH 3 so that the pH of the medium initially had to be adjusted to the value desired for the fermentation run. Fermentation runs under a specific set of conditions were repeated at least once, and in most cases, three replicate fermentations were performed. Data presented in the Figures and the text are the means of the replicates, and their standard deviations are represented by error bars in the Figures.

### Yeast conditioning and nutrition

The yeast (Ethanol Red™) employed for fermentations was a commercial dried yeast (Fermentis of Lesaffre, Milwaukee, WI, USA). Sufficient active dried yeast (0.5 g/L of fermentation medium) was rehydrated with the goal of providing a starting concentration of ~10^7 ^viable cells/ml in the fermentation medium. The yeasts were rehydrated/conditioned by first suspending into water an amount of Go-Ferm™ (Lallemand, Montreal, Canada) that equaled 1.25 parts by weight of that of the dried yeast that was to be used. The Go-Ferm™ was suspended into 20 times its weight of sterile H_2_O at 43°C. Once the temperature of the Go-Ferm suspension reached 39 to 40°C, the dried active yeast was added and gently stirred to evenly disperse the cells. The suspension was allowed to stand for 15 to 30 min during which time the yeast slurry cooled to ~32°C. The yeast slurry was then added to the feedstock (already at 32°C) in the fermentation vessel. Nitrogen supplementation was provided, when desired, by the addition of diammonium phosphate (DAP) (Sigma-Aldrich, St Louis, MO, USA) and/or yeast extract, HY-Yest 412 (10.2% total nitrogen; 5.2% amino nitrogen) (Sigma-Aldrich, St Louis, MO, USA). The yeast nutrient complex (Fermaid K™, Lallamand, Montreal, Canada), was routinely added at 0.25 g/L to the ongoing fermentation when the starting sugar levels were 1/3 depleted. For fermentations that utilized greater than 20% (w/v) sugars, yeast hulls were added at a level of 0.2 g/L to furnish additional sterols to the yeast and to adsorb part of the yeast autotoxins that are produced during fermentation.

### Yeast and bacterial counts

The density of viable yeast cells was routinely estimated by determining the absorbance at 420 nm of a diluted sample of fermentation medium. This relationship had been determined earlier by sampling, diluting, plating, and counting colony-forming units from fermentations that were in the log phase of growth. The number of colony-forming yeast cells per ml of media was then correlated with the absorbance at 420 nm of a sample from the fermentation medium measured at the same time as the sample was removed for plating. Microbial counts were taken from media after 48 h of fermentation. Dilutions of the fermentation medium were plated onto nutrient agar that contained 10 μg/ml of cyclohexamide (Sigma, St Louis, MO, USA) to inhibit yeast growth.

### Determination of ethanol and CO_2 _production rates

The rates of ethanol production at various pH values were estimated by plotting the ethanol levels versus fermentation times between 12 and 28 h and fitting the data with a linear least squares equation. The slope of the line was then used as a measure of the maximal rate of ethanol production. Although it is recognized that this type of direct plot is not truly linear, data in this region approach linearity (0.990 <*R*^2 ^< 0.999), and the slope of a linear fit to the data approaches the slope of a tangent to a point in this region.

The rate of CO_2 _evolution from the fermentor was estimated by counting the rate of bubbles released into the water trap per unit time. The conversion from number of bubbles per min to liters of CO_2 _per h was possible after first determining the number of CO_2 _bubbles from the water trap that it took to yield a liter of CO_2 _gas by displacement of water from an inverted water-filled graduated cylinder.

### Analytical methods

High performance liquid chromatography (HPLC) was carried out on a Varian ProStar ternary solvent system equipped with an autosampler and diode array and RI detectors. Quantitative carbohydrate profiles of the feedstocks were obtained with a 250 mm × 4 mm 5 μm Luna™ amino column (Phenomenex, Torrance, CA, USA). The sugars, glucose, fructose, and sucrose, were eluted with an isocratic system of 80% acetonitrile/20% H_2_O at a flow rate of 1 ml/min and a column temperature of 35°C. Fermentation substrates, glucose and fructose, and fermentation products, including ethanol, acetate, glycerol, citrate, and lactate, were separated and quantified on an Aminex™ HPX-87H 300 mm × 7.8 mm column (BioRad, Hercules, CA, USA). Components were eluted with an isocratic system of 5 mM H_2_SO_4 _in H_2_O. The flow rate was 0.6 ml/min, and the column temperature was maintained at 50°C. Three standard solutions containing fructose, glucose, and ethanol at different concentration levels were run each time a fermentation analysis was performed in order to confirm the fidelity of the respective calibration curves. In order to quantify fermentable sugars with the Aminex™ column during fermentation runs, samples that contained sucrose had to be pre-treated with invertase before column chromatography. This was necessary because the acid conditions of the 5 mM H_2_SO_4 _eluting solvent catalyzed hydrolysis of the glucose-fructose glycosidic bond during time on the column and created a reaction zone so that none of the three sugars could be quantified. Pre-column hydrolysis of sucrose with invertase was performed by incubating 100 μl of sample (up to 40% sucrose) with 100 μl of yeast invertase (Sigma, St Louis, MO, USA) at ~100 U/ml in 0.05 M sodium acetate buffer, pH 4.6, at 37°C for 30 min. By the end of the incubation, all sucrose had been hydrolyzed to glucose plus fructose, and these two hexose sugars could be separated and quantified by HPLC analysis on the Aminex™ column. Samples for analysis during fermentation were collected via the sterile sampler on the fermentor. Two 1 ml aliquots of the fermentation broth were centrifuged for 2 min at 10,000 × g on a microfuge (Eppendorf, Hamburg, Germany) to pellet all insoluble material. After invertase treatment and appropriate dilution (~50-fold), samples for HPLC analysis were filtered through 17 mm disposable nylon filters of 0.45 μm pore size before being placed in the autosampler. A sample loop of 100 μl was employed in the autosampler. Amino nitrogen was estimated in feedstocks by the ninhydrin method [9]. The concentrations of L-citrulline in the respective watermelon juices were estimated by separating the juice amino acids by thin layer chromatography [10] and comparing the intensity of each juice's L-citrulline spot, after staining with ninhydrin, to those of authentic L-citrulline co-developed on the chromatogram at known concentrations. The concentration of total nitrogen in a watermelon juice was estimated by measuring its amino nitrogen and adjusting for the additional nitrogen furnished by its L-citrulline content. This then assumed that all other amino acids in the watermelon juice each contained only one nitrogen. So while the estimate for amino nitrogen is accurate, the estimated numbers for watermelon juice total nitrogen may be as much as 5 to 15% lower than the true value since there was no quantitative measure of those amino acids that contained more than one nitrogen.

## Results and discussion

### Fermentation feedstocks

The watermelons employed in this study, their sources, and juice compositions are listed in Table 1. Total sugars among the watermelons ranged between 6.6 and 8.3% (w/v). Watermelon juice preparations from which the free amino acids had been removed contained ≤ 1 mmol of apparent amino N/ml but still possessed their original level of sugars. The molasses employed for fermentation contained 3.8% (w/v) glucose, 6.6% (w/v) fructose, 30.3% (w/v) sucrose, 42.0 mmol/ml of amino nitrogen by ninhydrin analysis, and ~14.2 g N/L (based on its published nitrogen content of 1.01% (w/w) of N).

**Table 1:** Feedstock watermelons employed and their respective juice compositions

**Watermelon source**	**Cultivar**	**(Glucose)** **(%, w/v)**	**(Fructose)** **(%, w/v)**	**(Sucrose)** **(%, w/v)**	**(Amino N)** **μmol/ml)**	**(Citrulline)** **μmol/ml)**
2007 Hinton, OK, USA	Crunchy Red	1.4	2.8	3.0	16.3	6.3

2007 Terral, OK, USA	Unknown	2.0	3.6	2.0	33.6	15.4

2008 Louisiana, USA	Summer Flavor 800	1.9	3.5	2.8	33.9	12.6

2008 Lane, OK, USA	Sangria	1.9	3.6	2.8	17.7	5.8

2008 Lane, OK, USA	Dixie Lee	1.7	3.1	1.8	19.9	3.7

Respective contents of amino nitrogen and concomitant citrulline varied among the watermelon juices. It is not known whether these differences are due to cultivar inherent differences or the result of variation in fertility and growing conditions. This can be indirectly illustrated by comparing the citrulline results for two of the cultivars that were used in this study with those for the same two cultivars that were also part of a study which compared citrulline levels in several watermelon cultivars [11]. The value for 'Sangria' flesh reported for the 2000 growing season was ~9.1 mmol/ml (1.6 mg/g flesh) while a citrulline content of 5.8 mmol/ml for 'Sangria' from the 2008 crop season was measured in the present study. Conversely, the value of ~6.8 mmol/ml (1.2 mg/g flesh) reported for citrulline in 'Summer Flavor 800' in 2000 is lower than 12.6 mmol/ml measured for citrulline in this cultivar in 2008.

### Fermentation of whole watermelon juice and as a waste stream from various processes

**Figure 1:**
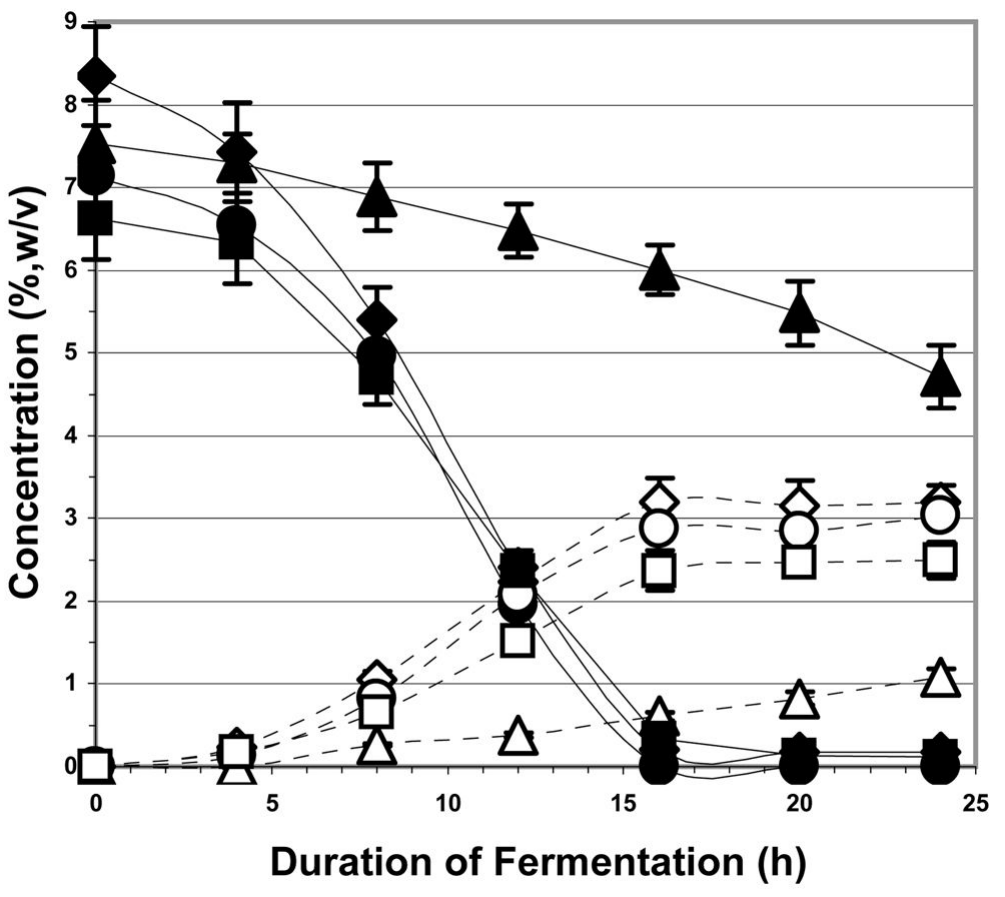
**The fermentation at pH 3 of watermelon juice and its partially processed states**. Closed diamonds = total fermentable sugars from whole watermelon juice of the 2008 Louisiana, USA crop; open diamonds = ethanol from whole watermelon juice of the 2008 Louisiana, USA crop; closed circles = total fermentable sugars from watermelon juice minus chromoplasts processed from the 2007 Hinton, OK, USA crop; open circles = ethanol from watermelon juice minus chromoplasts processed from the 2007 Hinton, OK, USA crop; closed triangles = total fermentable sugars from watermelon juice minus both chromoplasts and free amino acids processed from the 2007 Hinton, OK, USA crop; open triangles = ethanol from watermelon juice minus both chromoplasts and free amino acids processed from the 2007 Hinton, OK, USA crop; closed squares = total fermentable sugars from 2007 Hinton, OK, USA watermelon crop juice minus chromoplasts and free amino acids, but supplemented with 975 mg N/L; open squares = ethanol from 2007 Hinton, OK, USA watermelon crop juice minus chromoplasts and free amino acids, but supplemented with 975 mg N/L. Other conditions for the fermentations are described in the text.

Figure 1 illustrates the fermentation at pH 3 of watermelon juice from four different states: 1) whole watermelon juice; 2) watermelon juice with the lycopene-containing chromoplasts removed; 3) watermelon juice with the lycopene-containing chromoplasts and the free amino acids removed; and 4) watermelon juice with lycopene-containing chromoplasts and the free amino acids removed, but nitrogen-supplemented with yeast extract and DAP. Whole watermelon juice and juice with only the chromoplasts removed fermented at roughly the same rate and to the same extent. The yeast concentration started at 8 × 10^6 ^cells/ml and reached 6.0 × 10^7 ^cells/ml at 12 h. The rate of CO_2 _evolution at 12 h (fermentation was about 2/3 complete) was 3.2 L/h. In both cases, the sugar level of ~7% (w/v) was totally exhausted 16 to 20 h after inoculation with the yeast. The yield of ethanol from the complete watermelon juices averaged 0.41 ± 0.01 g/g sugars. Watermelon juice that had the free amino acids removed exhibited a much slower rate of fermentation. The total yeast available nitrogen in the media for this fermentation would have been less than 60 mg N/L. Starting with 8 × 10^6 ^cells/ml, the yeast concentration was 2.5 × 10^7 ^cells/ml and the CO_2 _evolution at 16 h was 0.6 L/h. Fermentation under these conditions took ~72 h to reach completion with a yield of 0.40 ± 0.02 g ethanol per g sugars. When watermelon juice with the amino acids removed was supplemented with yeast extract (295 mg N/L added before yeast addition; 295 mg N/L added at 6 h) and diammonium phosphate (106 mg N/L added before yeast addition; 233 mg N/L added at 6 h) for a total of 975 mg N/L, the fermentation rate returned to that of complete watermelon juice.

Lycopene-containing chromoplasts from the complete watermelon juice could still be solublilized in sodium dodecyl sulfate detergent (SDS) after 24 h in the fermentor at 32°C and in the presence of a final concentration of 3+% (w/v) ethanol. This SDS-solubility indicated that the integrity of the chromoplasts' membranes was still intact [12] so that the chromoplasts were still viable entities that could be isolated from the fermentation broth, if desired. Subsequently, chromoplasts from a fermentation that produced 12% (w/v) ethanol were also determined to be 97% viable. These results suggest that this neutraceutical could be successfully harvested intact post-fermentation and before removal of the ethanol by distillation.

### Watermelon juice as a nitrogen source for fermentation

The negative impact on fermentation rate by depletion of amino nitrogen from watermelon juice prompted an examination in more detail of the effect of nitrogen levels on fermentations involving this juice. Sucrose (table sugar) was employed as the primary feedstock. It was dissolved in chromoplast-free watermelon juice to a final sugar concentration of ~20% (w/v). This meant that the watermelon juice served as diluent, feedstock supplement (supplying ~70 g/L fermentable sugars to that of the total 200 g/L of sugars in the feedstock), and nitrogen source for the yeast. The level of free amino nitrogen in the fermentation media was varied by combining various proportions of amino acid-containing watermelon juice with amino acid-free watermelon juice. Nitrogen in these fermentations came almost exclusively from watermelon juice and varied from ~857 mg N/L down to ~46 mg N/L.

**Figure 2:**
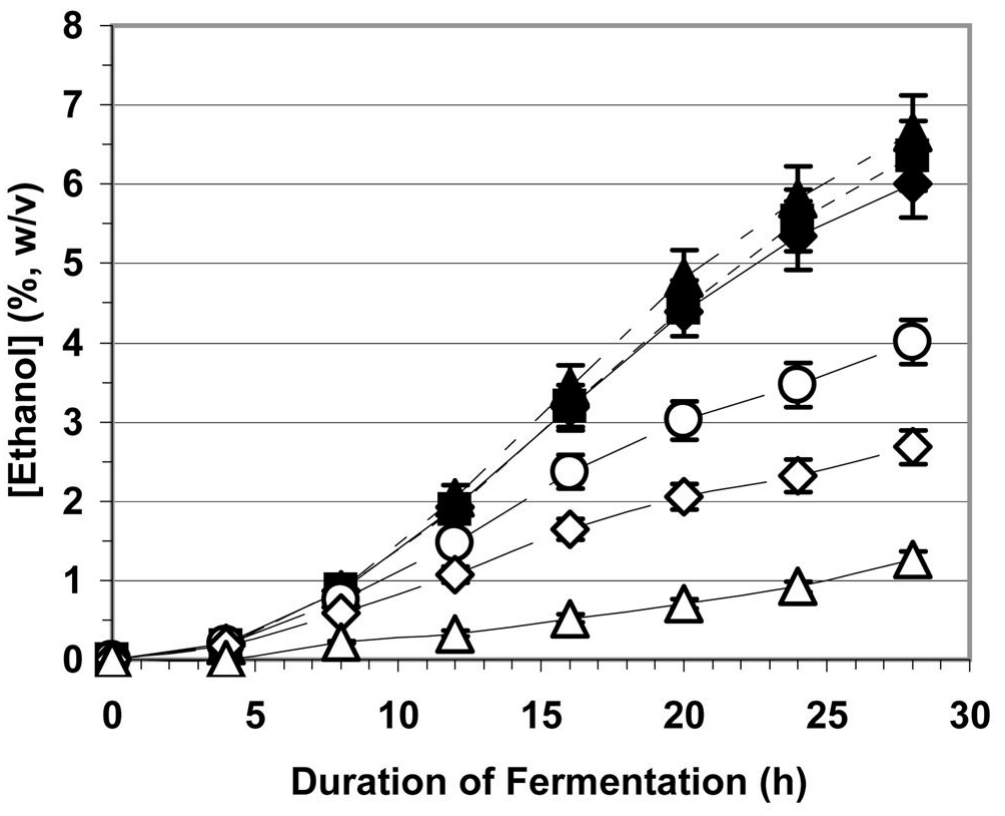
**The effect of yeast-available nitrogen on ethanol production rates by fermentation of sucrose/watermelon juice feedstocks at pH 3**. The initial concentration of total fermentable sugars in the fermentations was ~20% (w/v). Closed triangles = 857 mg N/L (33.6 μmol amino N/ml) from watermelon juice of 2007 Terral, OK, USA crop; closed squares = 890 mg N/L (33.9 μmol amino N/ml) from watermelon juice of 2008 Louisiana, USA crop; closed diamonds = 415 mg N/L (17.7 μmol amino N/ml) from watermelon juice of cv. 'Sangria', 2008; open circles = 230 mg N/L (10.6 μmol amino N/ml) from watermelon juice of cv. 'Sangria', 2008 from which part of the free amino acids had been removed; open diamonds = 138 mg N/L (6.7 μmol amino N/ml) from watermelon juice of cv. 'Sangria', 2008 from which part of the free amino acids had been removed; open triangles = 46 mg N/L (2.7 μmol amino N/ml) from watermelon juice of the 2007 Hinton, OK, USA crop from which the free amino acids had been removed. Other conditions for the fermentations are described in the text.

Figure 2 shows ethanol production over the first 28 h of fermentation at pH 3 of 20% (w/v) sugar solutions, each at a different level of nitrogen. When free amino acids from watermelon juice provided the nitrogen source, the rate of ethanol production was essentially constant above ~400 mg N/L (17 μmol/ml amino N). For these samples, fermentation was complete by 44 h and 0.48 ± 0.02 g ethanol was produced per g sugars. Below 400 mg N/L (or 17 μmol/ml amino N), the rate of ethanol production was proportional to the level of watermelon-source nitrogen. When nitrogen-free watermelon juice-sucrose feedstock was reconstituted to >400 mg N/L with yeast extract and DAP, the rate of ethanol production equaled that of the fermentation media of >400 mg N/L from watermelon juice (data not shown). Watermelon juice amino nitrogen thus appears to be as effective as the commonly employed nitrogen supplements for ethanol production by yeast fermentation and represents a potential cost-savings measure when employed as a diluent and nitrogen supplement for other feedstocks that lack or are low in available nitrogen. It can be inferred from these results that a split-stream strategy could be employed with watermelon juices that were well above the level of 400 mg N/L (17 μmol/ml of amino nitrogen) so that part of the juice would be utilized as a nitrogen source, diluent, and sugar supplement to provide maximal fermentation rates while the remainder would be used first for L-citrulline production and subsequently in ethanol production.

### Effect of pH on fermentation rates of feedstocks that include watermelon juice

**Figure 3:**
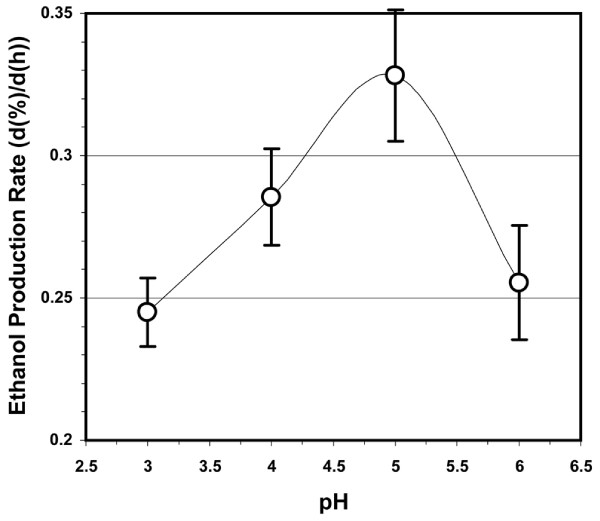
**The influence of the pH of fermentation on the rate of ethanol production from a combined sucrose/watermelon juice feedstock**. The initial concentrations of fermentable sugars were nominally 25% (w/v). The 2007 Hinton, OK, USA watermelon crop was the source of the juice. The juice was processed to remove chromoplasts before its use in the fermentations. The nitrogen content for all fermentations was 1,177 mg N/L (33.0 μmol amino N/ml). All fermentations were essentially completed (that is, all sugars consumed) after 48 h. Other conditions for the fermentations are given in the text, and the approach used to estimate *d*(%)/*d*(h) is given in *Methods*.

A series of solutions, each near 25% (w/v) in fermentable sugar concentration, were fermented by holding all variables constant except pH (Figure 3). The highest rate of ethanol production occurred near pH 5. The rate of ethanol production at this pH was roughly 30% greater than at pH 3 and pH 6. The optimal pH of 5 observed for this system is slightly lower than the pH of 5.73 utilized by Kim *et al*. [8] for their fermentations of watermelon juice. No significant differences were observed in the levels of ancillary metabolites produced in our system by the yeast strain employed (glycerol, citrate, lactate, and acetate) as the pH of the fermentation changed. The average yield of ethanol was observed to decrease slightly but consistently, from 0.39 ± 0.02 to 0.35 ± 0.02 g ethanol per g sugar, as the pH of the fermentation increased from pH 3 to pH 6. Forty-eight hour media plated from the runs at the various pH values yielded bacterial counts from 100 to 500 cfu/ml with no statistical difference among media samples at different pH values. Additionally, an anecdotal observation was that the spent fermentation medium exhibited a more noticeable fruity, flowery aroma at pH 5 and pH 6 than at pH 3 and pH 4. Because lower pH values are known to discourage bacterial growth, because some fermentation facilities may not possess means for narrow pH control of the fermentation, and because the pH of fermentations in watermelon juice was observed to quickly equilibrate at or near pH 3, subsequent experimental fermentations were conducted at or near pH 3 as well as at the apparent optimal pH near 5.

### Watermelon juice in the fermentation of cane sugar

Using watermelon juice as a media diluent, fermentable sugar supplement, and nitrogen supplement, various levels of cane sugar substrate were employed to determine conditions for the most efficient production of ethanol with our system (Table 2). When maintained at pH 3 and at the levels of nitrogen nominally furnished by watermelon juice (500 to 900 mg N/L), a starting feedstock concentration of about 25% (w/v) total fermentable sugars seemed to be the optimal starting level for the highest and most efficient ethanol production. At those starting concentrations of feedstock and nitrogen, virtually all of the feedstocks were fermented with a production of 0.43 ± 0.03 g ethanol per g sugar. With starting concentrations of sugar above 25% (w/v) at pH 3, the quantity of sugar that actually fermented maximized at around 25% (w/v). This left significant residual sugar and a final ethanol concentration that never rose above 10 to 12% (w/v). Conducting fermentations at pH 5 and supplementing the media with an additional 400 mg N/L (2.75 g/L yeast extract plus 0.5 g/L DAP) after 20 h of fermentation, allowed a utilization of up to 35% (w/v) total feedstock sugars at a production of 0.39 ± 0.02 g ethanol per g feedstock sugar.

**Table 2:** Fermentation of processed sugar supplemented and diluted with watermelon juice^a^

**Beginning (Fermentable sugars)** **(%, w/v)**	**pH**	**24 h (Yeast)** **(cells/ml)**	**24 h Respiration rate** **(CO_2_, L/h)**	**Final (Unfermented sugar)** **(%, w/v)**	**Final (Ethanol)** **(%, w/v)**	**Time to completion** **(h)**
18.3	3.1	6.8 × 10^7^	4.6	0	8.32	36

25.4	2.7	7.8 × 10^7^	3.8	0.4	11.6	86

29.3	2.8	5.1 × 10^7^	4.4	5.6	12.1	92

40.3	2.8	3.7 × 10^7^	2.8	18.2	10.2	93

40.3^b^	2.8			17.6^b^	10.5^b^	160^b^

26.9	5.0	11.0 × 10^7^	4.6	0.21	10.33	52

30.5	5.0	9.0 × 10^7^	4.4	0.18	11.53	68

40.95	5.0	7.9 × 10^7^	4.0	5.4	14.0	160

^a^Watermelon juices from the 2008 Louisiana crop and the 2007 and 2008 Oklahoma crops were employed. Chromoplasts were removed from the respective juices before their use in fermentation.

^b^This is the continuation of the fermentation presented immediately above; it demonstrates that little or no further ethanol production occurred.

### Watermelon juice as diluent, sugar supplement, and nitrogen supplement in the fermentation of molasses

**Figure 4:**
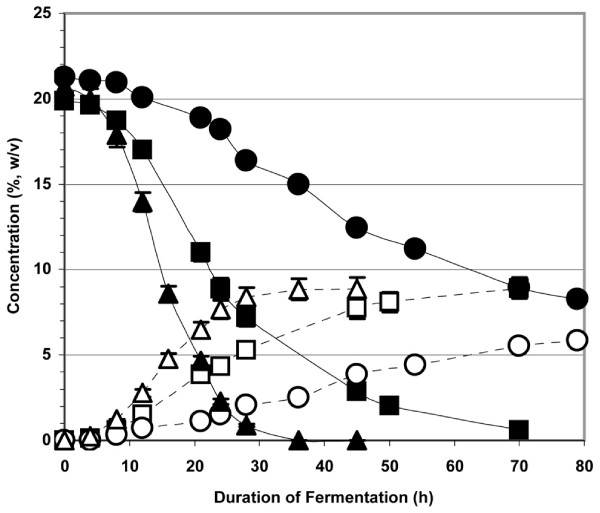
**The fermentation of molasses with watermelon juice used as both diluent and supplemental feedstock**. The 2007 Hinton, OK, USA watermelon crop was the source of the juice. Closed squares = sugar concentration of molasses diluted with watermelon juice minus chromoplasts. The nitrogen content for this fermentation was 385 mg N/L (6.1 μmol amino N/ml) from watermelon juice and 2066 mg N/L (9.2 μmol amino N/ml) from the molasses. Open squares = ethanol concentration produced from molasses diluted with watermelon juice minus chromoplasts; closed circles = sugar concentration of molasses diluted with chromoplast- and amino acid-free watermelon juice. The nitrogen content for this fermentation was 46 mg N/L (2.7 μmol amino N/ml) from the GoFerm™ and Fermaid K™ and 2066 mg N/L (9.2 μmol amino N/ml) from the molasses. Open circles = ethanol concentration produced from molasses diluted with chromoplast- and amino acid-free watermelon juice. Closed triangles = sugar concentration of molasses diluted with chromoplast- and amino acid-free watermelon juice that was nitrogen-supplemented with 500 mg N/L (22.5 μmol/ml amino N) from yeast extract (5.5 g/L), DAP (1.5 g/L), Go-Ferm™ (0.625 g/L), and Fermaid K™ (0.25 g/L) in addition to the nitrogen from the molasses. Open triangles = ethanol concentration produced from molasses diluted with chromoplast- and amino acid-free watermelon juice that was nitrogen-supplemented with 500 mg N/L in addition to that from the molasses. Other conditions for the fermentations are described in the text.

From earlier results, it was found that watermelon juice with the free amino acids removed requires another nitrogen source for the yeast. To that end, we examined the fermentation of molasses, diluted and sugar-supplemented using watermelon juice under three sets of conditions. The molasses was diluted approximately seven-fold with the watermelon juice to obtain the desired 20% (w/v) concentration of sugars. Watermelon juice provided ~400 mg N/L (6.1 μmol/ml amino N), GoFerm™ and Fermaid K™ provided 46 mg N/L (2.7 μmol/ml amino N), and molasses provided >2,000 mg N/L (8.4 μmol/ml amino N). Absent the nitrogen contributed by watermelon juice amino acids, the second fermentation medium contained only nitrogen contributed primarily by the molasses. A third fermentation was performed in which the molasses plus amino acid-free watermelon juice medium was supplemented with ~500 mg N/L (22.5 μmol/ml amino N) from yeast extract and DAP. The results presented in Figure 4 demonstrate the properties of the three systems. When the nitrogen source for the fermentation came almost exclusively from molasses (>2,000 mg N/L), the ethanol production rate was only about one-half the rate of fermentations that included amino acid-containing watermelon juice or yeast extract. Apparently, most of the nitrogen in molasses is not in a form readily assimilable by the yeast whereas the nitrogen source in watermelon juice is readily assimilated. The slight increase of fermentation rate in going from watermelon juice as the nitrogen source to yeast extract plus DAP as the nitrogen source was due to a pH difference in the runs: pH 2.9 versus pH 4.2. In a fermentation employing water as the diluent for molasses, nitrogen supplementation was also required to achieve ethanol production rates comparable to those that employed whole watermelon juice (data not shown). As was observed with refined sugar (Table 1), the molasses feedstock systems could completely and efficiently utilize up to ~35% (w/v) sugar concentration from the molasses at pH 5 and up to ~25% (w/v) sugar concentration at pH 3 with appropriate and adequate nitrogen supplementation.

### Applications of the technology

At a nominal total watermelon yield of 42 t/ha and 20% of the crop being culls, there would be about 8.4 t/ha of watermelons left in the field. A yield of 520 L juice/t containing 10% (w/v) fermentable sugars means the culls would produce 4370 L of juice possessing 435 kg fermentable sugars per ha. At a production ratio of ~0.4 g ethanol/g sugar as measured in this study, approximately 174 kg/ha or 220 L/ha of ethanol would be produced from these cull watermelons.

Two approaches exist for ultimately converting cull watermelons to ethanol. The most direct alternative would be to utilize the juice harvested from the culls to produce ethanol on-site at the production field with a mobile fermentation/distillation unit. Juice collected with a modified watermelon seed harvester would then only need to be transported from the rows of watermelon to the ethanol production system at the edge of the field. Using an ethanol production rate of 2 g/L/h of ethanol from watermelon juice as measured in this study (from Figures 1, 4, and Table 2), a required fermentation capacity can be calculated. The above approach would provide the grower with fuel for on-farm use and/or to sell on the ethanol fuel market.

In the second approach, watermelon juice in the form of a waste stream from neutraceuticals processing would be utilized as diluent and feedstock supplement in ethanol production. This scenario would likely involve a neutraceuticals production facility in physical proximity to an existing ethanol biofuel production facility. Watermelon production at a nominal rate of 42 t/ha has the potential for simultaneous production of 1.5 kg lycopene, 90 kg L-citrulline, and 2000 L ethanol per ha by utilizing both flesh and rinds. The removal of first lycopene-containing chromoplasts and then L-citrulline from watermelon has been developed into an integrated process that leaves a waste stream of sugar-containing juice (Fish, unpublished results). Both cull watermelons and those appropriate for the fresh food market are acceptable for this processing. Watermelon juice as a waste stream from neutraceutical production offers several advantages when integrated into a nearby bioethanol production facility. First, watermelon juice can be used in place of potable water as a diluent for the primary feedstock. Although wastewater treatment costs from post-fermentation waste would be little affected with either type of diluent, the use of a watermelon juice waste stream would save the cost of potable water at the front end of the process. Second, by using watermelon juice as diluent, the amount of primary feedstock required would be reduced by ~7 to 10% as a result of the readily fermentable sugars in the diluent juice. Based on the results using watermelon juice as a diluent for molasses fermentation, it is estimated that there would be an approximate 15% savings on molasses consumption and ~22,000 L savings of potable water per 40,000 L fermentation run. As well as serving as diluent and feedstock supplement, watermelon juice that retains its amino acids would also serve as a source of readily assimilable nitrogen for yeast growth and vitality and thus also save the costs associated with nitrogen supplementation.

## Conclusion

The results of this investigation indicate that watermelon juice as a source of readily fermentable sugars represents a heretofore untapped feedstock for ethanol biofuel production. The 8.4 t/ha of unmarketable watermelons left in the field at harvest would produce about 220 L/ha of ethanol for on-farm use or as an additional revenue stream for the grower. Whole watermelons utilized for the production of the neutraceuticals, lycopene and L-citrulline, yield a waste stream of sugar-containing juice. This juice waste stream would easily integrate with other more concentrated feedstocks employed in ethanol biofuel production where the watermelon juice would serve as diluent, supplemental feedstock, and nitrogen supplement.

## Abbreviations

SDS: sodium dodecyl sulfate.

## Competing interests

The authors declare that they have no competing interests.

## Authors' contributions

WF designed and coordinated the study and helped draft the manuscript. BB conceived the study and helped draft the manuscript. VR helped coordinate the study and helped draft the manuscript. All authors read and approved the final manuscript.
